# The Chain Mediating Effect of the Public's Online Health Information-Seeking Behavior on Doctor-Patient Interaction

**DOI:** 10.3389/fpubh.2022.874495

**Published:** 2022-06-02

**Authors:** Aijing Luo, Zhen Yu, Fei Liu, Wenzhao Xie

**Affiliations:** ^1^The Third Xiangya Hospital of Central South University, Changsha, China; ^2^The Second Xiangya Hospital of Central South University, Changsha, China; ^3^Key Laboratory of Medical Information Research, College of Hunan Province, Central South University, Changsha, China; ^4^School of Life Sciences, Central South University, Changsha, China

**Keywords:** online health information-seeking behavior, doctor-patient interaction, health belief model, e-health literacy, mediating effect

## Abstract

**Objective:**

This paper aims to explore the influence mechanisms of online health information-seeking behavior (OHISB) on doctor-patient interactions from a psychological perspective, using theory as a guide, which can effectively guide the mode of doctor-patient interaction after search behavior in China.

**Methods:**

We conducted a convenient web-based survey among members of the public who engage in searching behavior in China using a pretested structured questionnaire. Structural equation modeling was performed for path analysis and mediating effect testing.

**Results:**

The findings of the study show that (1) 4 control variables (education level, occupation, income, and diagnosed disease) had significant differences in online health information-seeking behavior; 7 control variables (age, gender, living area, education level, occupation, income, medical insurance) were significantly different in doctor-patient interaction behaviors. (2) perceived disease severity (95% CI: 0.003, 0.04, *P* < 0.001), perceived action benefits (95% CI: 0.059, 0.138, *P* < 0.001), and e-health literacy (95% CI: 0.061, 0.155, *P* < 0.001) were positive predictors between OHISB and doctor-patient interactions. (2) E-health literacy and perceived disease severity (95% CI: 0.001, 0.013, *P* < 0.05), and e-health literacy and perceived action benefits (95% CI: 0.082, 0.166, *P* < 0.001) play chain mediating roles between OHISB and doctor-patient interactions.

**Conclusions:**

E-health literacy, perceived disease severity, and perceived action benefits act as chain mediators between OHISB and doctor-patient interactions.

## Introduction

With the rapid development of China's economy, the material living conditions of the public are now satisfactory, and the public's health awareness is constantly improving. In recent years, with the rapid growth in users of the internet, the internet has become an important channel for the public to improve its health information literacy and gain access to health information (1). International research data have shown that the internet had become the main way for the public to obtain health information (2). According to the “Statistical Report on the Development of the internet in China,” as of June 2021, the population of internet users in China had reached 1.011 billion, an increase of 21.75 million internet users compared with December 2020, and the internet penetration rate had reached 71.6%. The number of people in China using the internet for medical purposes had the most significant growth, with 239 million users, accounting for 23.7% of all internet users (3). Internet medicine has effectively overcome temporal, spatial, and geographic restrictions, and it has continuously promoted the development process of medical digitization in China. The accelerated construction of 5G networks will bring great changes to the global medical big data industry chain and provide a sustainable guarantee for the public's sustainable access to health information.

Online health information-seeking behavior (OHISB) refers to behavior in which individuals seek information about health, risks, diseases, and health protection in the internet environment (4). It is believed that the more frequently patients engage in information-seeking behavior, the higher their health information level (5, 6) and the higher their participation in nursing management (7, 8). OHISB not only meets patients' health information needs but also improves users' electronic health (e-health) literacy (9). At the same time, however, OHISB may change the dynamics of the relationships between patients and health care providers (10). The reason for this is that internet health information lacks the relatively strict control that characterizes information shared on traditional media, and it coexists with offline hospitals that release authoritative health information, leading to the uneven quality of information (11, 12). Patients have difficulty obtaining recognition from doctors due to a lack of professional knowledge and background based on online judgment. Furthermore, there is an obvious digital gap between doctors and patients due to the patients' lack of professional knowledge (13, 14). The emergence of the internet has not been able to bridge the gap between doctors and patients; thus, doctor-patient interactions may be affected to some extent (15).

Doctor-patient interactions are the sum of the emotional interaction, psychological interaction, cognitive interaction, attitudinal interaction, and behavioral interaction between doctors and patients. From the perspective of maintaining patients' health, doctor-patient interactions should be harmonious; however, in recent years, the reality of changes in doctor-patient relationships has shown that the contradiction between doctors and patients has become prominent. Underlying this doctor-patient contradiction between doctors and patients has become prominent. The essence underlying the doctor-patient contradiction is the gap in health literacy between doctors and patients, which leads to a barrier to doctor-patient interactions (16).

The health belief model (HBM) is a social-cognitive model that explains and predicts people's health behaviors through their beliefs; it holds that strong health beliefs can lead to behavioral change in individuals (17). The knowledge, attitude, practice (KAP) model is the most common model used to explain the influence of personal knowledge and beliefs on health behavior; it holds that health literacy is the basis of health beliefs. Health beliefs are the power of health behavior, and health behavior is the product of the combination of knowledge and attitude (18). Although behavioral psychology has been widely studied, the impact of OHISB on doctor-patient interactions has rarely been assessed in psychology. Therefore, this study aims to explore the influence mechanisms of public OHISB on doctor-patient interactions from a psychological perspective, using theory as a guide. Therefore, this paper combines the HBM and the KAP model to predict and verify the paths of OHISB and doctor-patient interactions. In addition, control variables (age, gender, living area, education level, occupation, income, medical insurance, and diagnosed disease) were included in this research model to examine and adjust the effects of demographic factors on the research model.

## Materials and Methods

### Study Design

#### Determination of the Model Variables

The HBM consists of six factors: perceived susceptibility, perceived disease severity, perceived action benefits, perceived barriers to taking action, cues to action, and self-efficacy (19). The KAP model is the most commonly used model for explaining how personal knowledge and beliefs affect health behavioral change, and it emphasizes three continuous processes: knowledge, attitude, and practice. In this paper, the perceived disease severity, perceived action benefits, and e-health literacy (20) are selected to analyze the actual situation of OHISB.

Norman et al. (21) proposed the concept of e-health literacy and compiled the Electronic Health literacy Measurement Scale (eHEALS) in 2006. Guo et al. (22), domestic scholars, developed a Chinese scale of e-health literacy based on the items of the eHEALS according to the connotation of e-health literacy and foreign evaluation tools, which were used as a reference. The scale has good structural validity, content validity, and internal consistency. Through research, Champion (23) developed a reliable and effective scale for HBM testing, and the dimensions of perceived disease severity and the perceived action benefits are available and reliable based on scale testing. Based on the previous literature, scholars have not found a general and authoritative scale for the impact of OHISB on doctor-patient interactions. Anthony et al. (24), Australian scholars, developed a doctor-patient relationship scale related to health information seeking, and it includes the dimension of doctor-patient interactions. In this paper, the above scales were used to measure five variables: OHISB, e-health literacy, perceived disease severity, the perceived action benefits, and doctor-patient interactions. The summary of these variables is shown in Table 1.

**Table 1:** Research variables and reference sources.

**Variables**	**Conceptualization**	**Reference source**
Online health information-seeking behavior	The public's search for health information on the internet	-
Electronic health literacy	Individuals seek, understand and evaluate health information from electronic resources, and they process and use this health information to enhance their ability to solve health problems	eHEALS scales (22)
Perceived disease severity	The perceived severity of an individual's illness	(23)
Perceived action benefits	An individual's perception that a particular behavior can reduce disease damage or promote physical recovery	
Doctor-patient interaction	The interaction between the doctor and the patient	(24)

#### Determination of the Model Variables

To study the mechanism of the effect of OHISB on doctor-patient interactions, this paper discusses the mechanism of this effect based on interpretation-level theory. The mediating hypothesis is proposed from three dimensions, i.e., perceived disease severity, the perceived action benefits, and e-health literacy, and the following hypotheses regarding the relationship between e-health literacy and the HBM are proposed.

##### Mediating Role of Perceived Disease Severity

Perceived disease severity refers to the severity of a disease threat that an individual consciously suffers from, including the assessment of the disease's medical and social consequences (25). Harrison et al. (26) demonstrated that the more severe the perceived illness, the more likely people are to adopt healthy behaviors. When an individual suffers from a certain disease, after seeking health information, he or she will feel that the disease may threaten his or her life, bring pain or have an impact on his or her life. This feeling will not disappear or even be strengthened, which will lead to positive medical information-seeking behavior and increase the interaction with doctors when the individual faces doctors. Stanton et al. (27) confirmed that patients with a perceived disease threat tend to stay in touch with their doctor for a long time. Accordingly, the following hypotheses are proposed:

H1a: OHISB is positively associated with perceived disease severity.
H1b: Perceived disease severity is positively associated with doctor-patient interactions.
H1: Perceived disease severity plays a mediating role between OHISB and doctor-patient interactions.

##### Mediating Role of Perceived Action Benefits

Perceived action benefits refers to the perceived benefits of engaging in a healthy behavior to reduce disease damage or promote physical recovery. In this study, they refer to the public's perception of the benefits obtained from searching for health information on the internet. Willis (28) found that access to health information through online communities can improve the public's perceived benefits of disease treatment. Additionally, studies (29) showed that the more benefits the public perceived, the greater the promotion effect on people's adoption of healthy living behaviors. If the public perceives the benefits of self-health management through OHISB, they will be willing to express their views and have positive interactions with doctors when communicating with them. Accordingly, the following hypotheses are proposed:

H2a: OHISB is positively associated with perceived action benefits.
H2b: Perceived action benefits is positively associated with doctor-patient interactions.
H2: Perceived action benefits plays a mediating role between OHISB and doctor-patient interactions.

##### Mediating Role of Electronic Health Literacy

E-health literacy (21) refers to the ability of individuals to seek, understand and evaluate health information through online electronic media and to use the acquired health information to process and solve health problems to maintain and promote their own health. Keen (30) believed that OHISB can help improve the e-health literacy of inquirers, thus improving their disease prevention and health promotion abilities. Members of the public with high e-health literacy will reasonably analyze and judge the health information and health services suitable for them based on their own literacy. Neter and Brainin (31) believed that by using the same medical service, groups with high electronic information literacy would obtain more positive results in terms of health behaviors and health effects. Members of the public with higher e-health literacy can distinguish between real and false health information on the internet, while those with lower e-health literacy may believe false information on the internet, thus affecting the doctor-patient interactions process. According, the following hypotheses are proposed:

H3a: OHISB is positively associated with e-health literacy.
H3b: E-health literacy is positively associated with doctor-patient interactions.
H3: E-health literacy plays a mediating role between OHISB and doctor-patient interactions.

##### The Relationship Between Electronic Health Literacy, Perceived Disease Severity, and Perceived Action Benefits

Foreign studies have shown (32) that the degree of public knowledge of disease will have an impact on individuals' disease behavior. The higher the public's e-health literacy is, the higher people's ability to search for and collect health information through electronic media to perceive and predict their own symptoms in an accurate and timely manner (33). In other words, people with higher e-health literacy have a higher perceived disease severity and a better judgment of whether their OHISB will produce benefits. Accordingly, the following hypotheses are proposed:

H4a: E-health literacy is positively associated with perceived disease severity.
H4b: E-health literacy is positively associated with perceived action benefits.
H4: E-health literacy and perceived disease severity play a chain mediating role between OHISB and doctor-patient interactions.
H5: E-health literacy and perceived action benefits play a chain mediating role between OHISB and doctor-patient interactions.

#### Model Construction and Questionnaire Design

**Figure 1:**
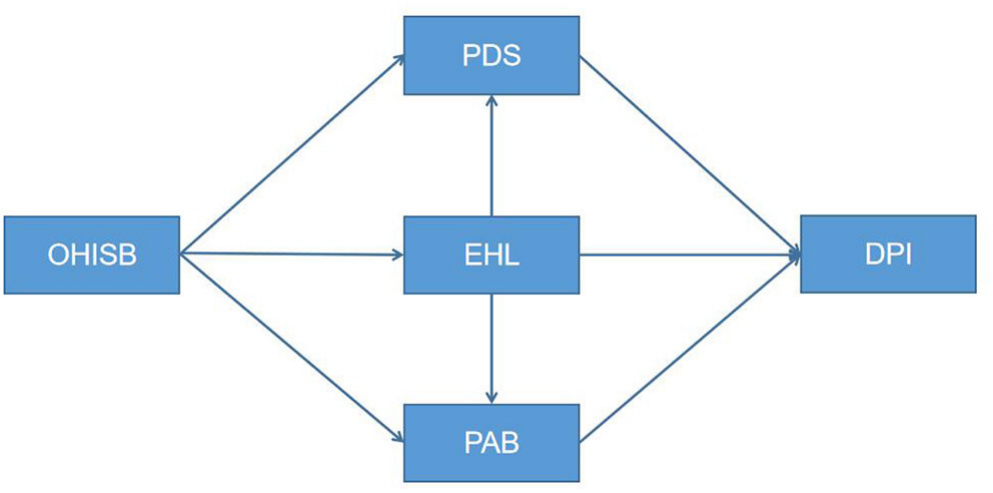
Schematic diagram of chained mediation model. OHISB, online health information-seeking behavior; PDS, perceived disease severity; PAB, perceived action benefits; EHL, electronic health literacy; DPI, doctor-patient interaction.

Based on the above research assumptions and theoretical framework, the OHISB-DPI mediation model is constructed, as shown in Figure 1. The questionnaire index pool was collected through a literature review, the initial questionnaire was formed through expert consultations and several rounds of revision by the research group, and the final questionnaire was formed by editing and text modification based on a preliminary experiment. The questionnaire is composed of five parts: a demographic information scale, an OHISB scale, a health belief scale, an e-health literacy scale, and a doctor-patient interactions scale. The respondents completed all parts using a 5-point Likert scale (strongly disagree, disagree, average, agree, strongly agree). Each variable and item of the questionnaire are shown in Table 2.

**Table 2:** Questionnaire variables and items.

**Variables**	**Codes**	**Items**
Online health information-seeking	A1	When I'm not feeling well, I turn to the internet for health information.
behavior (OHISB)	A2	When a relative or friend of mine has a disease, I will look up information about the disease on the internet.
	A3	When I'm worried about my health, I turn to the internet for health-related information.
	A4	I often look up health-related information on the internet.
Perceived disease severity	B1	I think that the thought of getting sick scares me.
	B2	I think that my career will be in jeopardy if I get sick.
	B3	I think that if I get sick, my financial security will be threatened.
Perceived action benefits	C1	I think that using the internet to find information can prevent some diseases.
	C2	Using the internet to find health information is conducive to recovery from illness.
	C3	Looking up health information helps me follow my doctor's advice.
Electronic health literacy	D1	I know how to find useful health information online.
	D2	I know how to use the internet to answer my health questions.
	D3	I know what kind of health information is available on the internet.
	D4	I know how to use the online health information I obtain to help myself.
Doctor-patient interaction	F1	Interactions with doctors have become more respectable as information is gathered from the Web.
	F2	Information on the internet helps me communicate more effectively with my doctor.
	F3	The information on the Web helps me ask my doctor smarter questions.
	F4	The information on the Web helps me better understand what my doctor tells me during consultations.

#### Data Source and Processing

##### Data Source

This study adopted the method of a network random questionnaire survey, relying on the design of Wengjuanxing, a questionnaire-making platform, and the questionnaire was delivered to the respondents through WeChat, QQ, and other network platforms. The participants' privacy was strictly protected. The data were collected in two stages. The first stage was the presurvey stage, during which reliability and validity analysis was conducted on the small sample data collected from 50 questionnaires. The second stage was a large-scale formal survey, with a total of 752 questionnaires collected. The data in this paper were based on strict questionnaire screening, and the elimination criteria were as follows: questionnaire samples whose completion time was <100 s were eliminated; questionnaire samples whose expressions were similar but whose answers were obviously contradictory were eliminated; questionnaire samples that did not have OHISB were eliminated. Finally, 713 valid questionnaires were obtained, for an effective response rate of 94.19%.

##### Data Processing

SSPS 23.0 and AMOS 23.0 were used for data processing, and the statistical analysis included descriptive statistics, structural equation model analysis, and bootstrap analysis. Descriptive statistics are expressed as the mean ± SD for quantitative data and frequency/percentage for categorical data. Independent samples *t*-test and one-way ANOVA were used to analyze the differences in online health information-seeking behavior and doctor-patient interaction among different populations.

### Statistical Analysis

#### Reliability and Validity Analysis

In this paper, the reliability of the items was tested by calculating the internal consistency coefficient (Cronbach's α). The validity test included content validity and structural validity, which were further divided into convergent validity and discriminant validity. Since all the scales in this paper were from mature scales in the existing literature, content validity was guaranteed. The convergent validity of the variables was tested through confirmatory factor analysis (CFA). The results obtained are shown in Tables 3, 4.

**Table 3:** Results of the reliability and convergent validity tests.

**Variables**	**Coding**	**Factor**	**Cronbach'α**	**CR**	**AVE**
		**loading**			
Online health	A1	0.897	0.951	0.952	0.832
information	A2	0.891			
seeking behavior	A3	0.957			
(OHISB)	A4	0.902			
Perceived disease	B1	0.719	0.836	0.844	0.644
severity	B2	0.888			
	B3	0.792			
Perceived action	C1	0.819	0.908	0.910	0.712
benefits	C2	0.923			
	C3	0.891			
Electronic health	D1	0.909	0.943	0.944	0.808
literacy	D2	0.917			
	D3	0.894			
	D4	0.874			
Doctor-patient	F1	0.727	0.918	0.921	0.747
interaction	F2	0.899			
	F3	0.921			
	F4	0.895			

*The factor load is the standard factor load; CR, Composite Reliability; AVE, Average Variance Extracted*.

**Table 4:** Results of the discriminant validity test.

**Main**	**OHISB**	**PDS**	**PAB**	**EHL**	**DPI**
**variables**					
OHISB	0.832				
PDS	0.297^***^	0.644			
PAB	0.418^***^	0.422^***^	0.712		
EHL	0.358^***^	0.261^***^	0.718^***^	0.808	
DPI	0.361^***^	0.354^***^	0.744^***^	0.670^***^	0.747
Square root	0.912	0.802	0.844	0.899	0.864
of AVE					

*** *Means P < 0.001. The diagonal data are AVE values. The data in lower triangle show the correlations between the variables calculated by AMOS software. AVE, Average Variance Extracted; OHISB, online health information-seeking behavior; PDS, perceived disease severity; PAB, perceived action benefits; EHL, electronic health literacy; DPI, doctor-patient interaction*.

##### Reliability Analysis

The results show that the Cronbach's α values of OHISB, perceived disease severity, the perceived action benefits, e-health literacy, and doctor-patient interactions ranged from 0.836 to 0.951 (as shown in Table 3). All values exceeded the standards proposed by Nunnally (34) and DeVellis (35). When the α coefficient is >0.8, the internal consistency of the questionnaire is considered excellent.

##### Validity Analysis

The results of the convergent validity test (as shown in Table 3) show that the standard load coefficients of all variables were >0.7 and had a high level of significance. Meanwhile, the composite reliability (CR) of each latent variable was >0.8, and the average variance extracted (AVE) was >0.6. The three criteria of convergent validity were satisfied, indicating that the convergent validity of the items was good. The results of the discriminant validity test (as shown in Table 4) show that the absolute values of the correlation coefficients between the latent variables ranged from 0.261 to 0.744. The square root of the AVE and correlation values were tested, and the values of the square root of the AVE were all larger than the correlation values, indicating good discriminant validity. In conclusion, the questionnaire had good reliability and validity.

#### Common Method Bias

To avoid artificial covariation between predictor variables and criterion variables caused by the same data source or rater, the same measurement environment, the project context or the characteristics of the project itself, this study adopted Harman's single-factor analysis to test for common method bias. The results show that the Kaiser-Meyer-Olkin (KMO) value = 0.915, *P* < 0.001, and the KMO value is >the 0.5 standard. Exploratory factor analysis revealed four factors explaining 79.002% of the variation in the total variance. Among them, the first factor explained 46.65% of the total variance, which was smaller than the judgment criterion of 50% determined by Podsakoff et al. (36).

#### Model Fitting

The analysis results of the structural equation model (as shown in Table 5) show that the χ2/df value was 3.569, which is <5. The value of the root means square error of approximation (RMSEA) was 0.06, which is <0.08. The value of the standardized root means square residual (SRMR) was 0.057, which is <0.08. The value of the goodness of fit index (GFI) was 0.932, which is >0.9. The value of the adjusted goodness of fit index (AGFI) was 0.909, which is >0.9. The value of the normed fit index (NFI) was 0.962, which is >0.9. The value of the Tucker-Lewis index (TLI) was 0.966, which is >0.9. The value of the comparative fit index (CFI) was 0.972, which is >0.9. In general, the fitting test results of the OHISB-DPI mediation model reached reasonable standards. The fitting degree of the model was good, and it could match well with the sample data.

**Table 5:** Model fit indexes.

**Index**	**χ ^**2**^/ df**	**RMSEA**	**SRMR**	**GFI**	**AGFI**	**NFI**	**TLI**	**CFI**
Observed value	3.569	0.060	0.057	0.932	0.909	0.962	0.966	0.972
Ideal value	<5	<0.08	<0.08	>0.9	>0.9	>0.9	>0.9	>0.9

*RMSEA is the root means square error of approximation; SRMR, standardized root means square residual; GFI, goodness of fit index; AGFI, adjusted goodness of fit index; NFI, normed fit index; TLI, Tucker-Lewis index; CFI, comparative fit index*.

## Results

### Descriptive Statistics

#### Demographic Characteristics

The descriptive statistics are shown in Table 6. The information related to the basic personal information of the respondents in this study mainly included their age, gender, place of residence, the highest level of educational attainment, occupation, monthly income, and type of medical insurance as well as whether they have suffered from clearly diagnosed diseases.

**Table 6:** Statistics of basic demographic characteristics.

**Items**	**Category**	***N***	**%**
Age	<18 years old	14	1.96%
	18–35 years old	432	60.59%
	36–59 years old	254	35.62%
	More than 60 years old	13	1.82%
Gender	Male	241	33.80%
	Female	472	66.20%
Place of residence	Urban	616	86.40%
	Rural	97	13.60%
Highest level of	Primary and below	11	1.54%
educational attainment	Junior high school	40	5.61%
	High school/technical secondary school	107	15.01%
	College	77	10.80%
	Undergraduate degree	255	35.76%
	Master's degree or above	223	31.28%
Occupation	Civil servant	33	4.63%
	Professional and technical personnel	143	20.06%
	Clerk	98	13.74%
	Enterprise management personnel	55	7.71%
	Workers	20	2.81%
	Farmers	18	2.52%
	Students	171	23.98%
	Freelancers	30	4.21%
	Self-employed	24	3.37%
	Unemployed	18	2.52%
	Retired (on-leave) personnel	14	1.96%
	Other	89	12.48%
Monthly income (CNY)	<3,000	220	30.86%
	3,000–5,999	226	31.70%
	6,000–8,999	104	14.59%
	More than 9,000	163	22.86%
Type of medical insurance	Medical insurance for urban workers	393	55.12%
	Medical insurance for urban residents (including college students)	184	25.81%
	The New Rural Cooperative Medical Care System	100	14.03%
	Other	21	2.95%
	No	15	2.10%
Clearly diagnosed	Yes	221	31.00%
diseases	No	492	69.00%

Based on the data collected in this study, the distribution of the demographic characteristics of the questionnaire survey can be understood. The respondents were mainly distributed among those belonging to the 18–35 age group (60.59%), followed by those belonging to the 36–59 age group (35.62%), and those belonging to the age groups of <18 years old and more than 60 years old. Female respondents accounted for 66.20%, and male respondents accounted for 33.80%. Most of the respondents lived in cities (86.40%). Most of the respondents (67%) had a university degree, i.e., a bachelor's degree or above. Among them, professional and technical personnel, staff, and students used the internet to search for health information, and those with a monthly income of more than 3000-5999 CNY accounted for 31.70% of the sample. The most common type of medical insurance was medical insurance for urban workers (55.12%), followed by medical insurance for urban residents (25.81%). The majority of the respondents did not have a clearly diagnosed disease (69%). Based on the results of descriptive statistical analysis, we analyzed the differences in online health information-seeking behavior and doctor-patient interaction behavior among different populations. The results in Tables 7, 8 showed that 4 control variables (education level, occupation, income, and diagnosed disease) had significant differences in online health information-seeking behavior; 7 control variables (age, gender, living area, education level, occupation, income, medical insurance) were significantly different in doctor-patient interaction behaviors. Specifically, younger patients, male patients, patients living in rural areas, patients with an education level of Primary and below, self-employed patients, and patients with a monthly income of <3,000 were more willing to interact with doctors; Patients with an education level of an undergraduate degree, self-employed patients, and patients with higher income, and patients with clearly diagnosed diseases were more likely to search for health information through the internet (Table 6). Statistics of basic demographic characteristics.

**Table 7:** Differential test of socio-demographic characteristics in online health information-seeking behavior (*N* = 713).

**Items**	**Category**	**Mean**	**SD**	***P*-value**
Highest Level of	Primary and below	2.8409	2.624	<0.001
educational attainment	Junior high school	3.2312	3.292	
	High school/technical secondary school	2.6238	1.384	
	College	3.2922	1.234	
	Undergraduate degree	3.4657	1.152	
	Master's degree or above	3.1502	1.325	
Occupation	Civil servant	3.606	1.099	<0.001
	Professional and technical personnel	3.455	1.154	
	Clerk	3.475	1.133	
	Enterprise management personnel	3.682	1.091	
	Workers	3.363	1.090	
	Farmers	2.944	1.052	
	Students	2.830	1.313	
	Freelancers	3.667	1.162	
	Self-employed	3.917	0.952	
	Unemployed	3.472	1.336	
	Retired (on-leave) personnel	3.375	1.251	
	Other	2.326	1.260	
Monthly income (CNY)	<3,000	2.865	1.280	<0.001
	3,000-5,999	3.011	1.294	
	6,000-8,999	3.541	1.097	
	More than 9,000	3.693	1.126	
Clearly diagnosed	Yes	3.499	1.277	<0.001
diseases	No	3.065	1.246	

**Table 8:** Differential test of sociodemographic characteristics in doctor-patient interactions (*N* = 713).

		**Mean**	**SD**	***P*-value**
Age	<18 years old	3.714	0.587	<0.001
	18–35 years old	3.44	0.651	
	36–59 years old	3.105	0.813	
	More than 60 years old	3.212	0.660	
Gender	Male	3.495	0.673	<0.001
	Female	3.234	0.744	
Place of Residence	Urban	3.299	0.739	<0.001
	Rural	3.474	0.664	
Highest level of	Primary and below	3.591	2.792	<0.001
educational attainment	Junior high school	3.419	0.748	
	High school/technical secondary school	2.792	0.895	
	College	3.114	0.760	
	Undergraduate degree	3.445	0.592	
	Master's degree or above	3.478	0.637	
Occupation	Civil servant	3.386	0.516	<0.001
	Professional and technical personnel	3.280	0.722	
	Clerk	3.388	0.622	
	Enterprise management personnel	3.418	0.585	
	Workers	3.400	0.825	
	Farmers	3.375	0.487	
	Students	3.554	0.622	
	Freelancers	3.542	0.550	
	Self-employed	3.271	0.780	
	Unemployed	3.653	0.637	
	Retired (on-leave) personnel	3.321	0.675	
	Other	2.635	0.873	
Monthly income (CNY)	<3,000	3.519	0.635	<0.001
	3,000–5,999	2.997	0.838	
	6,000–8,999	3.457	0.577	
	More than 9,000	3.422	0.626	
Type of medical insurance	Medical insurance for urban workers	3.192	0.762	<0.001
	Medical insurance for urban residents (including college students)	3.482	0.647	
	The new rural cooperative medical care system	3.533	0.680	
	Other	3.333	0.682	
	No	3.350	0.604	

#### Online Health Information-Seeking Behavior and Doctor-Patient Interaction Descriptive Statistics

Table 9 shows the mean and standard deviation of OHISB, perceived disease severity, perceived action benefits, e-health literacy, and doctor-patient interactions, as well as the Pearson correlation coefficients between variables. The correlations among all variables were significant, among which there were significant positive correlations among OHISB, perceived disease severity, perceived action benefits, e-health literacy, and doctor-patient interactions (*P* < 0.01).

**Table 9:** Correlations (*N* = 713).

**Main**	**M**	**SD**	**OHISB**	**PDS**	**PAB**	**EHL**	**DPI**
**variables**	**(Mean)**	**(Standard Deviation)**					
OHISB	3.20	1.27	1				
PDS	3.06	0.90	0.280^**^	1			
PAB	3.24	0.94	0.378^**^	0.391^**^	1		
EHL	3.38	0.83	0.335^**^	0.248^**^	0.674^**^	1	
DPI	3.32	0.73	0.322^**^	0.323^**^	0.684^**^	0.626^**^	1

** *Means P < 0.01. OHISB, online health information-seeking behavior; PDS, perceived disease severity; PAB, perceived action benefits; EHL, electronic health literacy; DPI, doctor-patient interaction*.

### Action Path Analysis

In this study, structural equation modeling in AMOS was used to calculate the standardized paths and data results. Based on the data results obtained, the influence relationship and significance of each variable were integrated into Table 10.

**Table 10:** Standardized path test results.

**Paths**	**Standardized coefficients (β)**	**S.E**.	**C.R**.	***P-*value**	**results**
H1a: OHISB → PDS	0.229	0.025	5.293	<0.001	support
H1b: PDS → DPI	0.077	0.025	2.549	0.011	support
H2a: OHISB → PAB	0.188	0.024	6.003	<0.001	support
H2b: 4 → DPI	0.514	0.03	10.827	<0.001	support
H3a: OHISB → EHL	0.359	0.024	9.46	<0.001	support
H3b: EHL → DPI	0.285	0.034	6.44	<0.001	support
H4a: EHL → PDS	0.188	0.039	4.356	<0.001	support
H4b: EHL → PAB	0.653	0.041	18.786	<0.001	support

*N = 713. OHISB, online health information-seeking behavior; PDS, perceived disease severity; PAB, perceived action benefits; EHL, electronic health literacy; DPI, doctor-patient interaction*.

There were 8 paths in this study, all of which were significant at the *P* < 0.05 level and could support the connectivity of the paths. OHISB affected perceived disease severity (β = 0.229, *P* < 0.001), perceived action benefits (β = 0.188, *P* < 0.001) and e-health literacy (β = 0.359, *P* < 0.001). e-health literacy affected perceived action benefits (β = 0.653, *P* < 0.001) and perceived disease severity (β = 0.188, *P* < 0.001). Doctor-patient interactions was affected by e-health literacy (β = 0.285, *P* < 0.001), perceived disease severity (β = 0.077, *P* < 0.05), and perceived action benefits (β = 0.514, *P* < 0.001). Therefore, H1a, H1b, H2a, H2b, H3a, H3b, H4a, and H4b are verified.

### Mediating Effect Analysis

**Figure 2:**
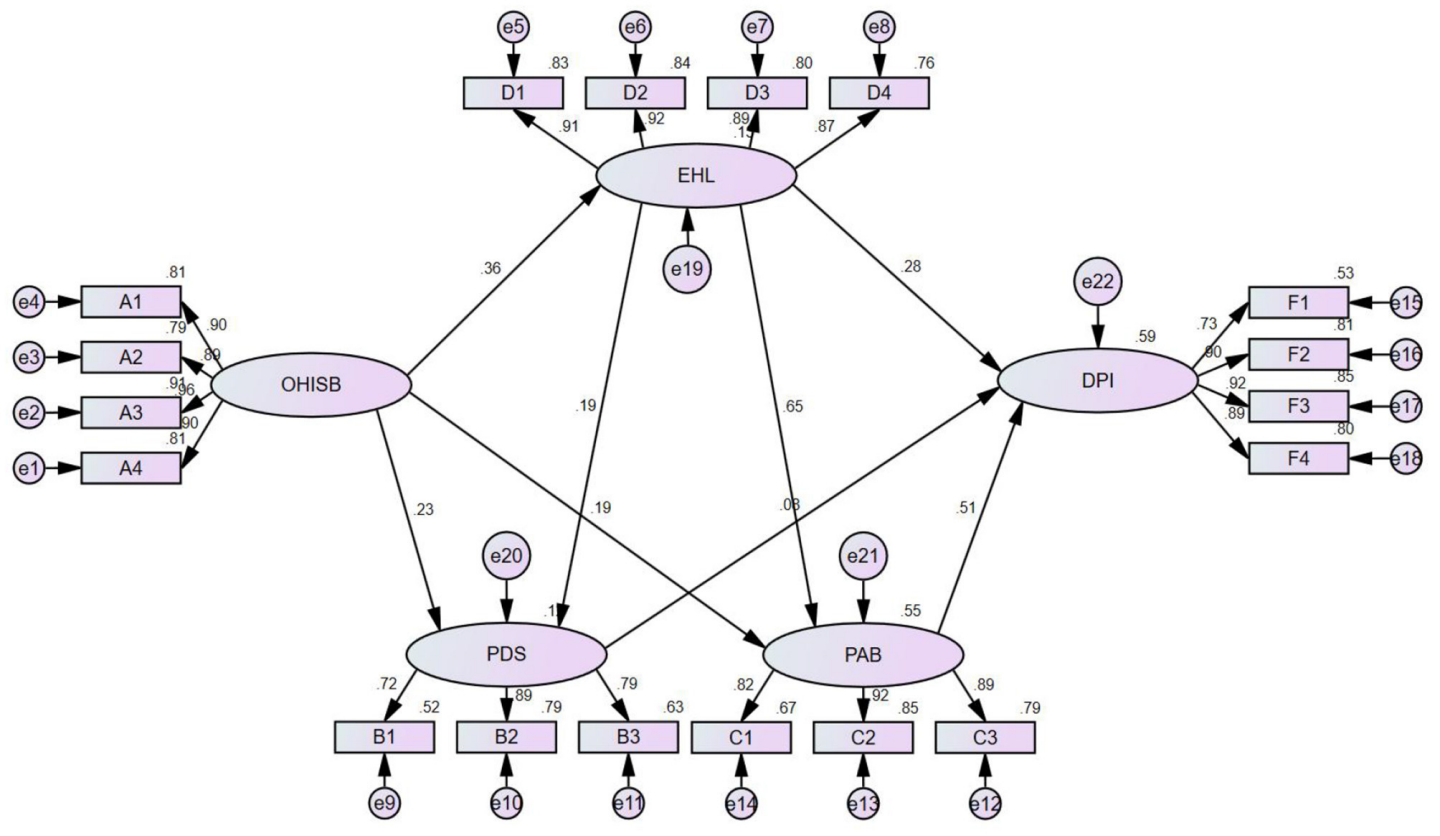
Standardized structural equation model. e1-e22 are the measurement errors of each variable, e.g., e1 is the measurement error of A4. A1-A4, B1-B3, C1-C3, D1-D4, F1-F4 are the observed variables of each latent variable, e.g., A1-A4 are the observed variables of OHISB. OHISB, PDS, PAB, EHL, DPI are the latent variables, i.e., they are the indicators of each dimension of this paper. OHISB, online health information-seeking behavior; PDS, perceived disease severity; PAB, perceived action benefits; EHL, electronic health literacy; DPI, doctor-patient interaction.

The bootstrap method was used for repeated sampling with 5000 iterations to conduct the mediating effect test (37). The mediating effect test and confidence interval (CI) estimation of the five paths were conducted, as shown in Table 11. The results show that the 95% CIs of the five paths did not include 0, indicating that the indirect effect was significant. Perceived disease severity (95% CI: 0.003, 0.04, *P* < 0.05), perceived action benefits (95% CI: 0.059, 0.138, *P* < 0.001) and e-health literacy (95% CI: 0.061, 0.155, *P* < 0.001) positively mediated the relationship between OHISB and doctor-patient interactions. The total standardized mediating effect value was 0.341, and the proportion of the indirect effect of the five paths in the total indirect effect was 5.3, 28.2, 29.9, 1.5, and 35.2%. Perceived disease severity (95% CI: 0.003, 0.04, *P* < 0.001), perceived action benefits (95% CI: 0.059, 0.138, *P* < 0.001), e-health literacy (95% CI: 0.061, 0.155, *P* < 0.001) fully mediated the relationship between OHISB and doctor-patient interactions. The e-health literacy and perceived disease severity (95% CI: 0.001, 0.013, *P* < 0.05) played the role of chain intermediary. And e-health literacy and perceived action benefits (95% CI: 0.082, 0.166, *P* < 0.001) also played the role of chain intermediary. H1, H2, H3, H4, and H5 are verified. The model is shown in Figure 2.

**Table 11:** Standardized bootstrap mediating effect.

**Mediation path**	**Standard estimate**	**Bias-corrected 95%** ***CI***			**Results**
		**Lower**	**Upper**	***P-*value**	
H1: OHISB → PDS → DPI	0.018 (5.3%)	0.003	0.04	0.014	Support
H2: OHISB → PAB → DPI	0.096 (28.2%)	0.059	0.138	<0.001	Support
H3: OHISB → EHL → DPI	0.102 (29.9%)	0.061	0.155	<0.001	Support
H4: OHISB → EHL → PDS → DPI	0.005 (1.5%)	0.001	0.013	0.011	Support
H5: OHISB → EHL → PAB → DPI	0.12 (35.2%)	0.082	0.166	<0.001	Support
Total indirect effect	0.341 (100%)	0.276	0.404	<0.001	Support

*The bias-corrected 95% CI lower limit and upper limit refer to the lower limit and upper limit of the 95% confidence interval for indirect effects estimated by the bias-corrected percentile bootstrap method, respectively. OHISB, online health information-seeking behavior; PDS, perceived disease severity; PAB, perceived action benefits; EHL, electronic health literacy; DPI, doctor-patient interaction*.

## Discussion And Conclusion

### Discussion

#### The Effect of OHISB on Health Beliefs and Knowledge and on Beliefs and Behavior

Currently, there are various search methods and rich health information resources on the internet (4). Due to convenient mobile devices, searching for health information is not restricted by time or space, and an increasing number of people are choosing to search for health information online (3). Previous research has shown an association between OHISB and the public's perceived disease severity, perceived action benefits, and e-health literacy (26, 28, 30). Based on the research results of this paper, OHISB has a positive impact on the public's perceived disease severity, perceived action benefits, and e-health literacy. Members of the public can correctly perceive the severity of the disease they are searching for, reasonably recognize the benefits brought by OHISB, and improve their e-health literacy related to the disease. The government can conduct free training courses on online health information-seeking skills so that the public can perceive the benefits of seeking health information online and acquire health information-seeking skills; at the same time, it should also strengthen the regulation of online information and educate the public on the use of websites that have been certified as high-quality information (38, 39).

#### OHISB Affects Doctor-Patient Interactions Through Public Health Beliefs

The results suggest that the public's perceived disease severity can serve as an intermediary between OHISB and doctor-patient interactions. Patients use more channels for health information when they are aware that their illness is potentially life-threatening (26), potentially disabling or painful, and that it will have an impact on their work or social role. This behavior may lead to the acquisition of mixed health information, which will impose more urgent requirements on the results of doctor-patient interactions (27). For the public, perceived action benefits can serve as an intermediary between OHISB and doctor-patient interactions. When patients perceive that searching for health information on the internet can prevent some diseases and discover the diseases they suffer from in a timely manner, they will actively and consciously interact with doctors and discuss the health information that they have obtained through their online searching with their doctors (28, 29). On this basis, the improvement in doctor-patient relationships has a relatively clear direction. Therefore, doctors should involve patients more in the medical decision-making process, so that patients can experience confidence in treatment in joint decision-making and reduce worries and anxiety brought about by fear of serious diseases; at the same time, hospitals can provide a reliable and authoritative platform for patients to obtain medical information. When the public passively receives pushing from a trusted platform, it will generate a sense of trust to enhance the perceived benefits of online health information-seeking behavior.

#### OHISB Affects the Public's Health Beliefs and Doctor-Patient Interactions Through the Public's E-Health Literacy

It was found that OHISB indirectly affected doctor-patient interactions through the chain mediating effect of e-health literacy and health beliefs. That is, the OHISB → e-health literacy → perceived disease severity → doctor-patient interactions path is established. Additionally, the OHISB → e-health literacy → perceived action benefits → doctor-patient interactions path is established. By searching for health information on the internet, members of the public can acquire health-related knowledge and improve their individual e-health literacy (21, 30, 31). Moreover, patients with different levels of eHealth literacy have different abilities to seek health information and judge information quality (40). When faced with a large amount of health information on the internet, members of the public with higher e-health literacy tend to behave more psychologically robustly. They will take the initiative to call on the existing health knowledge reserve, perceive the severity of the detected disease and perceive the convenient information service brought by the behavior of searching for their disease treatment, thus reducing the unequal knowledge background with doctors and promoting more confident interactions with doctors. Improving the public's e-health literacy requires the joint efforts of families, schools, and the government, such as education on general health knowledge in families and increasing health knowledge courses in schools. At the same time, government departments should also determine the evaluation criteria for e-health literacy. The current e-health literacy scale does not directly detect the public's health information-seeking skills.

## Conclusion

The results show that the HBM-KAP model is suitable for explaining the mechanism of the effect of the public's OHISB on doctor-patient interactions doctor-patient interactions. e-health literacy, perceived disease severity, and perceived action benefits act as a chain mediator between OHISB and doctor-patient interactions. In the era of the rapid development of the internet +, research on the functional path of OHISB and doctor-patient interactions can effectively guide the doctor-patient interactions mode and promote doctor-patient relationships in China. Members of the public can actively seek health information online, improve their own e-health literacy, and strengthen their personal health belief education to promote more effective doctor-patient interactions and create a harmonious medical environment. A harmonious medical environment also cannot be achieved without the joint efforts of the government, hospitals, and schools. For example, the government should strengthen the supervision of online health information distribution channels, hospitals should create a more authoritative medical information distribution platform, and schools should strengthen the training of health literacy.

### Practice Implications

Public online health information-seeking behavior has an impact on doctor-patient interaction, and this study aims to explore the mechanism of how patients' online health behavior affects doctor-patient interaction from the perspective of empathic mediation. It can enrich the theoretical study of the relationship between patient behavior and doctor-patient interaction and provide suggestions for improving patient cooperation by guiding the behavior of online physicians and patients, thus effectively alleviating doctor-patient conflicts and providing a reference for clinical practice in China.

## Data Availability Statement

The raw data supporting the conclusions of this article will be made available by the authors, without undue reservation.

## Ethics Statement

The studies involving human participants were reviewed and approved by the Third Xiangya Hospital, Central South University. The patients/participants provided their written informed consent to participate in this study.

## Author Contributions

AL conceptualized and designed the study as well as the investigation. AL and ZY drafted the original manuscript. FL designed the questionnaires together with AL and distributed questionnaires. ZY carried out the initial analyses and interpreted the data, review, and revised the manuscript. FL and WX distributed and collected questionnaires together. WX obtained funding for the research and supervised the procedure of the whole investigation evaluated the project. All authors contributed to the article and approved the submitted version.

## Funding

This study was supported by the Natural Science Foundation of Hunan Province (Grant No. 2021JJ70140).

## Conflict of Interest

The authors declare that the research was conducted in the absence of any commercial or financial relationships that could be construed as a potential conflict of interest.

## Publisher's Note

All claims expressed in this article are solely those of the authors and do not necessarily represent those of their affiliated organizations, or those of the publisher, the editors and the reviewers. Any product that may be evaluated in this article, or claim that may be made by its manufacturer, is not guaranteed or endorsed by the publisher.

## Abbreviations

DPI: Doctor-patient interaction
EHL: Electronic health literacy
HBM: Health belief model
KAP: Knowledge, attitude practice model
OHISB: Online health information-seeking behavior
PAB: Perceived action benefits
PDS: Perceived disease severity.
